# Chronic Kidney Disease and Disproportionally Increased Cardiovascular Damage: Does Oxidative Stress Explain the Burden?

**DOI:** 10.1155/2017/9036450

**Published:** 2017-11-23

**Authors:** Anila Duni, Vassilios Liakopoulos, Karolos-Pavlos Rapsomanikis, Evangelia Dounousi

**Affiliations:** ^1^Department of Nephrology, Medical School of the University of Ioannina, Ioannina, Greece; ^2^Division of Nephrology and Hypertension, 1st Department of Internal Medicine, AHEPA Hospital, School of Medicine, Aristotle University of Thessaloniki, Thessaloniki, Greece

## Abstract

Chronic kidney disease (CKD) patients are among the groups at the highest risk for cardiovascular disease and significantly shortened remaining lifespan. CKD enhances oxidative stress in the organism with ensuing cardiovascular damage. Oxidative stress in uremia is the consequence of higher reactive oxygen species (ROS) production, whereas attenuated clearance of pro-oxidant substances and impaired antioxidant defenses play a complementary role. The pathophysiological mechanism underlying the increased ROS production in CKD is at least partly mediated by upregulation of the intrarenal angiotensin system. Enhanced oxidative stress in the setting of the uremic milieu promotes enzymatic modification of circulating lipids and lipoproteins, protein carbamylation, endothelial dysfunction via disruption of nitric oxide (NO) pathways, and activation of inflammation, thus accelerating atherosclerosis. Left ventricular hypertrophy (LVH) and heart failure are hallmarks of CKD. NADPH oxidase activation, xanthine oxidase, mitochondrial dysfunction, and NO-ROS are the main oxidative pathways leading to LVH and the cardiorenal syndrome. Finally, a subset of antioxidant enzymes, the paraoxonases (PON), deserves special attention due to abundant clinical evidence accumulated regarding reduced serum PON1 activity in CKD as a contributor to the increased burden of cardiovascular disease. Future, meticulously designed studies are needed to assess the effects of antioxidant therapy on patients with CKD.

## 1. Introduction

Chronic kidney disease (CKD) patients are among the groups at the highest risk for cardiovascular disease. There is abundant evidence in the literature regarding the inverse association between kidney function and cardiovascular risk [1–4]. Premature cardiovascular disease extends from mild to moderate stages of CKD. Cardiovascular mortality is about twice as high in patients with stage 3 CKD (estimated glomerular filtration rate (eGFR) 30–59 mL/min per 1.73 m^2^) and three times higher at stage 4 (15–29 mL/min per 1.73 m^2^) compared to individuals with normal kidney function. Strikingly, in patients with mild to moderate CKD, the incidence of cardiovascular mortality is much higher than the incidence of end-stage renal disease (ESRD) [3]. Patients with CKD and end-stage renal disease (ESRD) carry a 5- to 10-fold higher risk for developing cardiovascular disease compared to age-matched controls [4]. According to the United States Renal Data System (USRDS) report, the expected remaining lifespan of patients while on maintenance dialysis was approximately eight years for patients 40 to 44 years of age and approximately 4.5 years for those 60 to 64 years of age suggesting that survival especially in older patients is comparable only to that of patients with lung cancer [5]. Considering the fact that CKD is at the spotlight as an urgent public health concern with a growth rate of 8% annually, it is imperative to elucidate culprit pathophysiological mechanisms of cardiovascular disease in CKD so as to come up with measures towards improved patient outcomes [6, 7].

The spectrum of cardiovascular disease in the CKD population extends from arterial vascular disease including atherosclerosis and arteriosclerosis to left ventricular remodeling with concentric hypertrophy, left ventricular dilation, and subsequent heart failure, with nonatherosclerotic cardiovascular disease becoming dominant at more advanced stages of CKD [8]. Traditional cardiovascular risk factors, such as hypertension and diabetes, can only partly explain the severely increased cardiovascular burden in patients with CKD [9]. Complex pathophysiological processes, otherwise referred as the nontraditional CKD-related risk factors, have been suggested to being etiologically involved in the pathogenesis of cardiovascular disease in this patient group [9, 10]. Oxidative stress and a chronic inflammatory state are among others at the center of attention as possible novel cardiovascular risk factors with direct pathogenic implications in CKD [8–11].

Increased oxidative stress in CKD, similarly to other pro-oxidant states, is characterized by excessive generation of oxidant compounds due to activation and upregulation of various enzymatic pathways including nicotinamide adenine dinucleotide phosphate (NADPH) oxidase, lipoxygenase, xanthine oxidase, uncoupled nitric oxide synthase (NOS), and the mitochondrial respiratory chain [12, 13]. Insufficient antioxidant defense mechanisms, both the enzymatic and nonenzymatic ones, further amplify this state of enhanced oxidative stress in CKD. Superoxide dismutase (SOD), catalase, and selenium-containing glutathione peroxidase have been traditionally considered as the main mediators of the enzymatic antioxidant protective pathway [14]. Additionally, another family of enzymes, the paraoxonases (PON), which share distinct properties, has increasingly received much attention due to its potential discrete albeit substantial role in the antioxidant armamentarium of the organism [15].

The aim of this review is to describe the available links between experimental and clinical evidence regarding the intricate mechanisms via which CKD enhances oxidative stress in the organism with ensuing cardiovascular damage and as a consequence amplification of cardiovascular morbidity and mortality. Similar backgrounds are required so as to find common pathophysiological denominators which shall serve as future targets of therapy.

## 2. Pathways of Oxidative Stress in CKD

Progressive renal dysfunction is characterized by amplification of oxidative stress. This has been indicated by several clinical studies which showed a gradual augmentation of levels of different oxidative stress markers including plasma F2-isoprostanes, advanced oxidation protein products, and malonyldialdehyde (MAD) as well as oxidized LDL in CKD patients in parallel with advancing stages of renal dysfunction [12, 13, 16, 17]. Oxidative stress in patients with renal disease has been ascribed to the effects of processes specifically linked to the loss of renal function and/or the modality of renal replacement therapy. In particular, oxidative stress in uremia is supposed to be mainly the consequence of higher ROS production, whereas attenuated clearance of pro-oxidant substances due to renal dysfunction as well as impaired antioxidant defenses seem to play a complementary role. It has been suggested that oxidative stress itself accelerates renal injury progression via cytotoxic mechanisms, thus perpetuating a vicious circle. Plasma ROS levels reflect in fact local ROS production in the kidneys, and among others, NADPH oxidases (NOX) with Nox4 are the major sources of ROS in the kidneys [17]. The increase in oxidative stress is most prominent in ESRD patients, with longer durations of dialysis therapy being associated with further elevation of oxidative stress markers [13, 16].

The pathophysiological mechanism underlying the increased ROS production which is invariably present in all forms of CKD is at least partly mediated by the pathological upregulation of the intrarenal angiotensin system, as evidenced by marked upregulation of angiotensin II receptors (AT1 and AT2) and simultaneous increases in angiotensin II-producing cells in the diseased kidney. It has been shown that activation of the AT1 receptor by angiotensin II increases superoxide production in the kidney and vasculature via NOX isoforms [18, 19]. Specifically, Nox4, predominant isoform in the renal tissue and the mitochondrial respiratory chain, has been implicated in the basal production of ROS in the kidney tissue and upregulation of Nox4 has been associated with specific pathologic conditions such as diabetic nephropathy and CKD progression [20].

Enhanced oxidative stress in the setting of the uremic milieu modifies circulating lipids and lipoproteins causing essential alterations of their biological properties. Thus, covalent modifications of lipids and lipoproteins generate several lipid end products such oxidized phospholipids (oxPLs), fatty acid peroxidation products (FAPPs), oxysterols, and F2-isoprostanes [21]. Lipid peroxidation byproducts such as MDA are increased and are negatively correlated with GFR in CKD. MDA covalently binds to proteins and nucleic acids, interfering with their normal biological functions [21]. In CKD, several markers have emerged as well-suited indicators of oxidative stress-related atherosclerosis: MDA, F2 isoprostanes, lipid hydroperoxides, asymmetric dimethylarginine (ADMA), 8-oxo-7,8-dihydro-2′-deoxyguanosine, protein carbonyls, advanced oxidation protein products, and glutathione-related activity [22, 23]. Particularly, ADMA, an inhibitor of NOS, has been shown as a strong and independent predictor of cardiovascular outcome and mortality in patients with advanced CKD. Although ADMA level is regulated by its renal clearance, it has been proposed that the activity of the enzyme dimethylarginine dimethylaminohydrolase that regulates the generation of ADMA is sensitive to oxidative stress, thus providing evidence for the crosstalk that exists between oxidative stress and endothelial dysfunction [14, 24].

Patients with kidney disease and most markedly ESRD patients have been shown to display not only higher rates of pro-oxidant activity but impairment of antioxidant defenses as well [12]. Nuclear factor-erythroid-2-related factor 2 (Nrf2) is a key molecule involved in the coordinated induction of over several genes, including the ones associated with encoding antioxidant and detoxifying enzymes and related proteins. Experimental studies have shown that CKD animals exhibited a marked and time-dependent decline in nuclear Nrf2 content, despite the presence of oxidative stress and inflammation, which should have induced Nrf2 activation and upregulation of its target genes [25, 26].

Furthermore CKD is usually associated with the presence of other pro-oxidant states such as diabetes mellitus, dyslipidemia, hypertension, and aging. Hyperglycemia and advanced glycation end products (AGEs) promote ROS generation by the mesangial cells. AGEs are a heterogeneous group of compounds which are derived from the nonenzymatic glycation of proteins, lipids, and nuclear acids through a complex sequence of reactions. They are produced not only in the presence of hyperglycemia but also in the setting of increased oxidative stress, as it occurs in CKD [27]. Nox4 and uncoupled NOS are markedly upregulated in diabetic nephropathy, which together with impairment of mitochondrial metabolism are the main sources of ROS generation in the kidney and vascular tissues [27–31]. Arterial hypertension, both a cause and consequence of CKD, is associated with amplified oxidative stress, subsequent endothelial dysfunction, increased systemic vascular resistance, renal sodium retention, and further exacerbation of arterial pressure. Indeed, hypertension is well known to be a major state of oxidative stress as in almost all experimental models of hypertension, ROS are increased in multiple organs, including critical centers of the brain, the vasculature, and the kidney [32]. In the kidney, ROS act in multiple sites to promote sodium resorption and volume retention whereas in blood vessels, ROS promote vasoconstriction and remodeling, increasing systemic vascular resistance [32]. Upregulation of NADPH oxidase via the activation of renin-angiotensin system (RAS) and dysfunctional mitochondria is a well-defined major provider of ROS in hypertension [32–34]. There is also emerging evidence that ROS contribute to immune activation in hypertension. A recent experimental study showed that byproducts of fatty acid oxidation react with protein lysines to form oxidized pyrrole adducts. These act as autoantigens that are presented to T lymphocytes by antigen presenting cells, leading to T cell activation. Activated T cells then infiltrate the vascular wall and release cytokines which in turn induce collagen deposition, thus causing aortic stiffening, renal inflammation, and ultimately hypertension [35].

Inflammation is the most common outcome of oxidative stress, and both processes are deeply interrelated in kidney diseases in a vicious cycle as both amplify each other [11, 36]. Oxidative stress directly activates transcription factors such as NF-*κ*B, which regulate expression of gene proinflammatory cytokines and chemokines [37]. Likewise, oxidative stress promotes recruitment and activation of leukocytes, formation of proinflammatory oxidized lipids, and advanced protein oxidation products and AGEs. On the other hand, activated leukocytes, macrophages, and resident cells generate ROS, chlorine, and nitrogen species with further augmentation of oxidative stress [38]. Accordingly, free radical generation by NADPH oxidase has been found to be abnormally increased in circulating lymphocytes and monocytes from patients from even the early stages of CKD [38].

## 3. Vascular Oxidative Stress Implications in Atherosclerosis

The process of atherogenesis involves the interplay of oxidized plasma lipids and inflammatory cells of the immune system within a dysfunctional vascular wall. The elucidation of the intricate mechanisms through which the pro-oxidant systems promote atherogenesis has been the subject of extensive and ongoing research and is beyond the scope of our review.

The major ROS-producing systems expressed in the vascular wall, with some of them being expressed by the immune cells as well, include the Nox1- and Nox2-reduced isoforms of NADPH, xanthine oxidase, uncoupled endothelial NOS (eNOS), and enzymes of the mitochondrial respiratory chain [39]. The superoxide anion which is generated in the vascular wall via all the aforementioned systems is converted to hydrogen peroxide by the antioxidant enzyme SOD [40]. Nevertheless, hydrogen peroxide may as well spontaneously convert to the hydroxyl radical which is extremely reactive to most cellular components [41, 42]. A number of findings suggest that augmented superoxide anion levels play a substantial part in the pathogenesis of atherosclerosis. Nitric oxide is produced by a family of NOS with three NOS isoforms identified including neuronal NOS (nNOS), inducible NOS (iNOS), and eNOS. Apart from NO, NOS also produces superoxide anion, a phenomenon known as NOS uncoupling. Although superoxide generation mainly occurs when NOS is not coupled with its cofactor or substrate as it occurs under L-arginine depletion condition, it has been suggested that superoxide generation is an intrinsic process amid NO synthesis [39]. Accordingly, the superoxide anion may directly inactivate NO or promote oxidation of the endogenous NOS cofactor tetrahydrobiopterin, leading to NOS uncoupling with decreased NO production, thus inducing endothelial dysfunction [43]. Decreased NO production due to changes in the expression and activity of eNOS and increased degradation of NO, by reaction with superoxide account for the reduction in endothelium-dependent vascular relaxation [41]. Additionally, peroxynitrite, which is produced via the reaction between the superoxide anion and NO, is a strong oxidant of proteins, lipids, and nucleic acids thus resulting in further vascular damage [44]. Finally, the superoxide anion facilitates oxidative modification LDL lipoproteins that play a key role in the formation of atherosclerotic lesions [42, 43].

Several protective antioxidant mechanisms such as glutathione, peroxidase, and catalase are available within the vascular wall in order to detoxify hydrogen peroxide [39–42]. eNOS is considered an atheroprotecive enzyme as it produces NO, which induces vascular smooth muscle relaxation while inhibiting its proliferation, platelet aggregation, leukocyte adhesion to the vascular endothelium, and oxidation of LDL particles. eNOS may become dysfunctional in the setting of a pro-oxidant environment [45, 46].

Classical risk factors for atherosclerosis are considered to induce the pro-oxidant pathways as well as eNOS uncoupling in the vascular wall thus creating a vicious circle where oxidative stress begets more oxidative stress. Accordingly, increased oxidative stress is directly linked to endothelial dysfunction by reducing ΝΟ availability with subsequent alterations in the vascular permeability and entrance of LDL cholesterol within the vascular intima where it is oxidized as well as to migration of inflammatory cells in the subendothelial space after expression of adhesion molecules by the dysfunctional endothelium [45, 46].

There is abundant experimental evidence available, which has shed light on the implications of oxidative stress, chronic inflammation, and endothelial dysfunction as well as their crosstalk in the pathogenesis of atherosclerosis [40, 47]. Nevertheless, there are not as plenty clinical data and especially longitudinal studies which directly correlate oxidative stress with atherosclerotic cardiovascular disease and its outcomes in patients with CKD. Most probably, this should be ascribed to the lack of a strict and uniform definition of oxidative stress markers as well as to the large range of techniques used for their determination and the numerous confounding factors in CKD which make it difficult to interpret the results of these studies. As direct in vivo detection of ROS is technically difficult, most studies are based on the detection of end products of redox chemical reactions which indirectly estimate the in vivo level of oxidative stress. Thus, biomarkers of oxidative stress are either molecules that are modified by interactions with ROS or molecules of the antioxidant system which are altered in response to increased oxidative stress. Examples include modified nucleic acids, lipids, proteins, and carbohydrates which serve as biomarkers in clinical studies [48].

## 4. Oxidative Stress and Accelerated Atherosclerosis in CKD

**Figure 1:**
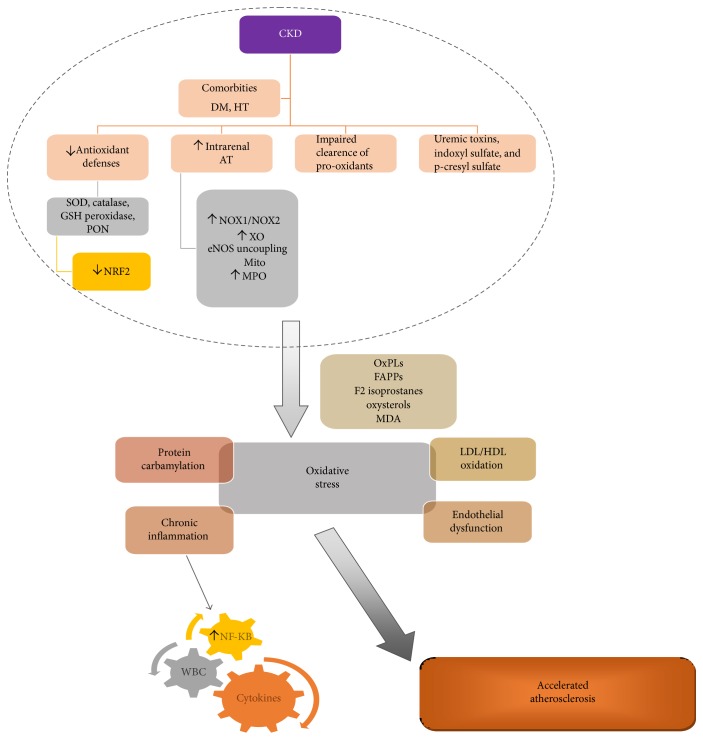
Pathways of amplified oxidative stress in CKD and byproducts leading to accelerated atherosclerosis. AT: angiotensin; DM: diabetes mellitus; eNOS: endothelial nitric oxide synthase; FAPPs: fatty acid peroxidation products; GSH: glutathione; HT: hypertension; MDA: malondialdehyde; Mito: mitochondria; MPO: myeloperoxidase; NF-*κ*B: nuclear factor kappa-light-chain-enhancer of activated B cells; NOX: NADPH oxidase; NRF2: nuclear factor E2-related factor 2; OxPLs: oxidized phospholipids; PON: paraoxonase; SOD: superoxide dismutase; WBC: white blood cells; XO: xanthine oxidase.

The specific processes via which CKD-associated oxidative stress promotes accelerated atherogenesis have been extensively studied (Figure 1). The enzyme myeloperoxidase (MPO) and its derived oxidants have been for long subject of research, as mediators of oxidative modification of biomolecules and tissues. MPO uses hydrogen peroxide to oxidize chloride to the strong-oxidizing agent hypochlorous acid, a toxic agent to various biomolecules such as lipoproteins and the eNOS substrate l-arginine [49]. Thus, MPO promotes LDL modifications through various pathways such as the reaction between HOCl generated by MPO and tyrosine residues of ApoB100, generation of reactive nitrogen species (RNS) resulting in proatherogenic nitrosilated LDL, and MPO-catalyzed addition of thiocyanate to the LDL leading to the formation of carbamylated LDL (cLDL) [50]. Additionally, MPO can oxidize NO to nitrite thus abolishing its protective properties on the vascular wall [49]. As MPO is released in the circulation during chronic inflammation, its measurement in systemic circulation may be used as an index of leukocyte activation and oxidant stress. MPO levels correlate with angiographic evidence of coronary atherosclerosis and cardiovascular events in subjects with chest pain within the general population [51–53]. Increased MPO levels have been found to be associated with complex and calcified atherosclerotic lesions and incident cardiovascular disease. In patients with end-stage CKD undergoing hemodialysis, serum MPO levels have been found to correlate with oxLDL, with levels of markers of inflammation and prospective mortality risk [54–56]. Interestingly, in hemodialysis patients, MPO activity has been associated with aortic stenosis as well [57].

Οxidated lipids are important players in the initiation and progression of atherosclerotic changes. The oxidation of LDL by HOCl generated in MPO-catalyzed reaction is thought to be a proatherogenic event which precedes the formation of foam cells, a hallmark of atherosclerotic plaque development. Oxidized epitopes of LDL can initiate an immune response and lead to the formation of antibodies directed against oxLDL. Both OxLDL and antibodies against oxLDL have been correlated with carotid atherosclerosis and cardiovascular events in HD patients [58, 59]. High levels of oxidized HDL are correlated to increased cardiovascular mortality in HD patients [60]. HDL is a major target for oxidative stress being subjected to posttranslational modifications. Generation of dysfunctional HDL through MPO-mediated oxidative damage to apolipoprotein A-1 (ApoA1), the major HDL protein, is associated with inability of HDL to remove cellular cholesterol by the ATP-binding cassette transporter A1 (ABCA1) pathway in humans with atherosclerosis. As a consequence, as MPO activity is enhanced in CKD, MPO-modified ApoA1 results in decreased reverse cholesterol efflux. Furthermore, MPO leads to the increased formation of an oxidation product—3-chlorotyrosine—in HDL and impairs lecithin-cholesterol acyl-transferase (LCAT) and paraoxonase (PON) activities and thus the anti-inflammatory properties of HDL. MPO-modified HDL particles are also potentially involved in the generation of foam cells in atherosclerotic lesions, increased expression of adhesion molecules, and impaired antiapoptotic properties in endothelial cells [61–65].

A recent clinical trial showed that renal transplantation does not correct impairment of uremic HDL particles. Accordingly, HDL cholesterol acceptor capacity and antioxidative activity were profoundly suppressed in kidney transplant recipients independent of graft function and were comparable with levels in patients with ESRD, suggesting that pathologic modification of HDL may contribute to the residual cardiovascular risk in the transplant population [66]. The cardioprotective effect of HDL is mainly attributed to its cholesterol efflux capacity, which was shown to inversely correlate with atherosclerotic cardiovascular disease in populations with normal kidney function. Nevertheless it should be noted that as demonstrated by a recent clinical trial, HDL cholesterol efflux capacity might not be a prognostic cardiovascular risk marker in patients with diabetes on hemodialysis [66, 67].

Carbamylation, which is a posttranslational modification of proteins or amino acids, results either from nonenzymatic reactions of amino acid residues with urea decomposition products such as isocyanic acid, or through a MPO-catalyzed pathway. Recently, it has been shown that carbamylated LDL induces endothelial dysfunction and increased ROS production, leading to eNOS uncoupling [68]. Additionally, carbamylated LDL possesses proatherogenic properties, such as promoting foam cell formation via interaction with macrophage scavenger receptors as well and vascular smooth muscle proliferation [69, 70]. On the other hand, HDL loses its antiapoptotic activity after carbamylation, contributing to endothelial cell death [71]. Protein carbamylation levels have emerged as a particularly strong predictor of both prevalent and incident cardiovascular disease risk [68–72].

In subjects with angiographic evidence of coronary artery disease, peripheral artery disease, myocardial infarction, stroke, or previous revascularization, protein carbamylation as assessed by plasma protein-bound homocitrulline level was found significantly higher than healthy controls. It should be noted that in clinical studies, protein-bound homocitrulline levels predicted cardiovascular disease even after extensive adjustments for traditional cardiovascular risk factors, renal function, and both MPO levels and high sensitivity C-reactive protein concentrations [73]. The plasma level of protein-bound homocitrulline has been found to predict increased cardiovascular risk in patients with ESRD as well, thus further supporting the interplay and crosstalk among uremia, inflammation, and atherosclerosis mechanisms [74, 75]. In patients with CKD, including patients with ESRD undergoing hemodialysis, clinical studies have shown that elevated carbamylated LDL concentrations are associated with significantly higher all-cause mortality, as well as shorter cardiovascular event-free survival [76–78].

Indoxyl sulfate, one of the uremic toxins associated with accelerated progression of CKD, has been suggested to induce vascular disease via amplification of oxidative stress. Experimental studies have shown that indoxyl sulfate stimulates proliferation of rat vascular smooth muscle cells [79]. Indoxyl sulfate upregulates the expression of intercellular adhesion molecule-1 (ICAM-1) and monocyte chemotactic protein-1 (MCP-1), a process mediated via ROS-induced activation of NADPH oxidase and nuclear factor-*κ*B (NF-*κ*B) in the vascular endothelial cells [80]. Subsequently, indoxyl sulfate is suggested to play an important role in the development of cardiovascular disease in CKD patients by increasing the endothelial expression of ICAM-1 and MCP-1 [81–83]. However, a recent meta-analysis regarding the association of indoxyl sulfate and p-cresyl sulfate with cardiovascular events and all-cause mortality in patients with CKD showed that elevated levels of both uremic toxins are associated with increased mortality in these patients, but only p-cresyl sulfate was associated with an increased risk of cardiovascular events [84].

Clinical research has aimed at providing unifying models of oxidative stress markers in CKD. In a recent study, antioxidant defenses were measured together with markers of oxidative stress and inflammation markers in patients with CKD experiencing cardiovascular distress and were compared to normal age and sex-matched individuals. Levels of SOD, catalase, and glutathione as well as NO levels were decreased in patients with CKD whereas MDA levels and IL-1 levels were increased, further supporting the fact that impairment of antioxidative capacity of the organism accompanied by a proinflammatory state is associated with cardiovascular risk in CKD [85].

Coronary artery calcifications, a hallmark of the atherosclerotic burden, are a ubiquitous finding in patients with advanced stages of CKD and more so in ESRD [86]. Vascular calcification requires vascular smooth muscle cell (VSMC) differentiation into osteoblast-like cells [86]. Increased intracellular oxidative stress shown increase osteoblastic differentiation of vascular and bone cells in vitro, thus modulating differentiation of vascular cells [87, 88]. In a recent experiment in male rats with CKD, vascular calcification was induced with a high calcium-phosphate diet and vitamin D supplementation [87]. Thoracic aorta was subsequently harvested for assessment of vascular calcification, macrophage infiltration, cytokine expression, vascular smooth muscle differentiation, ROS generation, and related signaling pathway activation. The expression of interleukin-1*β*, interleukin-6, and tumor necrosis factor (TNF) were increased in the calcified lesions of the aortic media as were the expression of NADPH oxidase subunits whereas the expression of antioxidant enzymes was reduced in CKD high-calcium-phosphate diet rats. Additionally, oxidized peroxiredoxin, a sensor of ROS generation, was significantly increased and ROS-sensitive signaling pathways were activated in the aorta from CKD high-calcium-phosphate diet rats [87]. Few clinical studies have assessed the relationship between biomarkers of oxidative stress and coronary artery calcifications. MDA levels and lipid peroxides were shown to increase with increasing coronary artery calcium score [89–93]. Additionally, the serum MDA-modified LDL/LDL cholesterol ratio was shown to be significantly higher in hemodialysis patients than in nondialysis subjects and has been independently associated with the coronary artery calcium score [94].

Increased common carotid artery intima-media thickness is considered an early phase of atherosclerosis. It has been associated with cardiovascular risk and risk of coronary artery disease events and has been largely used as a marker of atherosclerosis for clinical studies. Cross-sectional studies which have investigated the possible association between biomarkers of oxidative stress and intima-media thickness of the carotid artery in CKD patients have shown controversial results [95–102].

Similarly, the clinical studies which have evaluated possible association between oxidative stress and the prevalence of cardiovascular disease in hemodialysis patients yielded conflicting results as well [103–109]. However, it would be beyond the scope of this review to discuss each of them in detail. Overall, a significant correlation between biomarkers of oxidative stress and prevalence of cardiovascular disease was demonstrated in most studies. MDA serum level is higher in hemodialysis patients with ischemic heart disease, stroke, cerebrovascular disease, and peripheral vascular disease and to be an independent predictor of cardiovascular disease as well [106]. Additionally, serum MDA levels in hemodialysis patients correlated with the severity of cardiovascular disease and the cardiac index as well [107, 108]. Finally, most studies, which sought to correlate biomarkers of oxidative stress with cardiovascular events, cardiovascular-related mortality, and all-cause mortality in patients undergoing renal replacement therapy, found a positive association as well [56, 106, 110–117].

Nevertheless, the efficacy of antioxidant therapy in terms of prevention of cardiovascular disease in people with CKD remains controversial. A large meta-analysis included approximately 2000 dialysis and nondialysis CKD patients and kidney transplant recipients treated with various antioxidants, including different doses of vitamin E, coenzyme Q, acetylcysteine, bardoxolone methyl, human recombinant SOD, and multiple antioxidant therapy [118]. Compared with placebo, antioxidant therapy showed no clear benefit with regard to cardiovascular mortality, all-cause mortality, coronary heart disease, cerebrovascular disease, or peripheral vascular disease. However, significant benefit was conferred by antioxidant therapy for cardiovascular disease prevention in dialysis patients but not in CKD patients whereas antioxidant therapy was found to significantly reduce incidence of ESRD [118]. It has been suggested that the failure of antioxidants utilized in clinical trials most probably should be ascribed to their inability to target specific enzymatic pathways directly involved in ROS generation. Accordingly, results from these trials indicate that oxidative injury associated with CKD and CVD would most probably be hindered by agents targeting the causal pathway of oxidative injury.

## 5. Oxidative Stress, Left Ventricular Hypertrophy, and the Cardiorenal Syndrome

The prevalence of left ventricular hypertrophy (LVH) significantly increases with deterioration of CKD stages [119]. Thus, prevalence of LVH may be up to 31% in individuals with CKD stage III and it increases to more than 50% in predialysis patients, reaching 90% after the initiation of renal replacement therapy [120, 121]. The main pathological features of left ventricular remodeling are myocardial apoptosis and intramyocardial fibrosis which lead to decreased myocardial capillary density, impaired diastolic filling of the ventricle and contractile dysfunction, and eventually chamber dilation [122, 123]. The severity and persistence of LVH are strongly associated with mortality risk and cardiovascular events in CKD and ESRD patients [124]. Additionally, abnormal impulse conduction as it occurs with LVH might account for the greatly increased prevalence of sudden cardiac death in CKD (59 deaths per 1000 person yearly) compared to the general population (1 death per 1000 person yearly) [121, 125]. Although increased vascular stiffness, volume overload, and anemia are considered as the major determinants of LVH in CKD, inappropriate activation of the renin-angiotensin-aldosterone system (RAAS), oxidative stress, and inflammation are the potential mediators implicated in its pathogenesis [120, 122, 123].

The relatively recent term cardiorenal syndrome has been applied as a unifying concept to the bidirectional interaction between heart disease and kidney disease as either acute or chronic dysfunction of the one organ can, respectively, cause acute or chronic dysfunction in the other [6, 126]. Specifically, type 4 cardiorenal syndrome is classified as CKD contributing to cardiac hypertrophy, depressed cardiac function, and subsequently increased risk of adverse cardiovascular events.

NADPH oxidase activation, xanthine oxidase, mitochondrial dysfunction, and NO-ROS are the main pathways through which increased oxidative stress is generated in CKD leading to LVH and cardiorenal syndrome [127–130]. While, at low concentrations, ROS modulate important physiological functions through changes in cellular signaling and gene expression, overproduction of ROS may adversely alter cardiac mechanics, leading to further worsening of systolic and diastolic function. Abundant experimental and clinical data have linked increased oxidative stress to the development of LVH, a process mediated by the activation of several promitogenic kinases, transcription factors and matrix metalloproteinases, promotion of cardiac fibroblast proliferation, and amplification of the hypertrophic stimulus of angiotensin II to the myocardium [127, 131]. Thus, ROS activate a broad variety of hypertrophy signaling kinases and transcription factors, such as MAP kinase (MAPK) and nuclear factor-*κ*B (NF-*κ*B) and they play an important role in G protein-coupled hypertrophic stimulation by angiotensin II [127, 132]. In addition, ROS stimulate overexpression of cellular apoptosis signaling kinase-1, which activates NF-*κ*B to stimulate hypertrophy [133]. ROS also promote extracellular matrix accumulation, through stimulation of cardiac fibroblast proliferation and activation of matrix metalloproteinases (MMPs) [127, 131].

Accordingly, a growing body of evidence suggests that in the setting of heart failure (HF), ROS production within the myocardium and the vasculature is substantially increased [133]. ROS depress cardiomyocyte contractility through damage of myofibrillar proteins with ensuing decreased calcium sensitivity [134]. Moreover, mitochondrial ROS intervene with intracellular signaling by triggering the transcriptional activation of specific nuclear genes [134–136]. Finally, high ROS concentrations activate stress kinases like c-Jun *N*-terminal kinase (JNK) and p38-mitogen-activated MAPK promoting both cardiomyocyte autophagy and mitochondrial autophagy, eventually leading to apoptosis [134–136]. Chronic activation of RAAS in HF and CKD stimulates inflammation, apoptosis, fibrosis, and oxidative stress in both the heart and the kidney [136, 137]. Angiotensin II activates cardiac and renal NADPH oxidase and subsequently production of ROS, which in turn stimulate the production of proinflammatory mediators among which interleukin-6 and TGF-*β* thus contributing to cardiac and renal fibrosis [138–140]. In addition, excessive sympathetic activity induces hypertrophy of cultured cardiomyocytes via superoxide anion O_2_^−^ production and has a growth-promoting effect on the wall of renal vasculature mediated by ROS production [141, 142].

The chronic inflammatory state, present in both CKD and HF, is associated with increased levels of proinflammatory cytokines such as IL-1b, IL-6, and TNF-*α*, which in experimental models have been shown to further stimulate angiotensin II-mediated ROS production in cultured rat aortic smooth muscle cells [143, 144].

Vascular endothelial dysfunction due to uncoupling of the NOS, activation of vascular and phagocytic membrane oxidases, or mitochondrial oxidative stress may lead to increased vascular stiffness, further compromising cardiac performance in the failing myocardium [145]. The normal coupled eNOS pathway is thought to provide an inhibitory influence on hypertrophy and hypertrophic signaling, MMP activation, and cardiac dysfunction [145]. The excessively produced NO derived from NOS has also been implicated in the pathogenesis of chronic HF as the combination of NO and superoxide yields peroxynitrite, a reactive oxidant, which has been shown to impair cardiac function [145].

Heart failure is associated with a shift in energy metabolism form *β*-oxidation of fatty acids to glycolysis; thus, free fatty acids are not transported into the mitochondria and accumulate into the cytosol, thus activating the oxidative cascade [146]. Pathological alterations of the mitochondrial electron transport chain as the main source of ROS have become at the center of attention in HF models [147–149].

There is a lack of clinical studies regarding direct correlation of oxidative stress markers and LVH in patients with CKD. However, a recent clinical trial, conducted in children with CKD, showed a positive correlation between serum levels of oxidized LDL and protein carbonyl group and left ventricular mass [150].

Likewise, despite the abundant experimental evidence regarding the interplay of different oxidative stress pathways in HF and CKD, there is a relative paucity of clinical research regarding this issue. A recent study conducted in diabetic patients with end-stage CKD undergoing hemodialysis showed that serum carbamylated albumin was strongly associated with the 4-year risk of death from congestive HF, thus suggesting a direct link between carbamylation and uremic cardiomyopathy in patients with diabetes mellitus and kidney disease [151]. Most clinical studies so far have focused on the search of specific therapies which aim to reduce oxidative stress. So far, RAAS blockade, *β*-blockers, and statins have been shown to reduce the burden of oxidative stress in the organism whereas most clinical trials which utilized administration of specific antioxidants vitamin C and vitamin E failed to show a benefit for the treatment of cardiovascular diseases, including HF [133, 152]. Novel therapies which target selective inhibition of NADPH oxidases or mitochondrial biogenesis are the subject of ongoing research [153–155]. Finally, supplementation with coenzyme Q10 has been suggested to exert a beneficial antioxidant role both in the kidneys and in the heart with improved clinical outcomes in patients with the cardiorenal syndrome [156–159].

## 6. The Defense System: Alteration of Paraoxonases in CKD and Cardiovascular Implications

The paraoxonases (PON), another family of enzymes with shared distinct properties, has increasingly been the subject of extensive investigation due to its potential discrete albeit substantial role in the antioxidant mechanism of the organism. Thus, we believe that considering the substantial clinical evidence accumulated, the PON merit special attention.

The PON family includes 3 enzymes, PON1, PON2, and PON3, which belong to the armamentarium of the antioxidant defenses of the organism, with PON1 being the most extensively studied [15]. They are coded by adjacent genes located on chromosome 7q21-22 with several polymorphisms being described both in the coding and the promoter regions of the PON genes, with direct effects on enzyme concentrations and activities [15]. PON1 and PON3 are found in the circulation associated with HDL as well as in tissues, whereas PON2 location is intracellular, associated with the endoplasmic reticulum and the nucleus [15]. Human paraoxonase/arylesterase 1—PON1 is a glycoprotein possessing lactonase and peroxidase property. Although the exact mechanism of PON1 action as well as its specific endogenous substrate has not been clarified yet, experimental data have shown that its spectrum of activity encompasses from hydrolyzation of lipophilic lactones to degradation of oxidized lipids. A possible physiological substrate is homocysteine thiolactone, which is a known risk factor in atherosclerosis, because metabolic conversion of homocysteine to thiolactone and protein homocysteinylation by thiolactone may play a role in homocysteine-induced vascular damage. PON1 associates with HDL in the circulation and exerts some of its systemic antioxidant activities, via preventing lipoprotein oxidation, degrading hydrogen peroxide and lipid peroxides, and promoting cholesterol efflux from macrophages and hydrolyzation of homocysteine thiolactone [15, 160, 161]. PON1 has been shown to inhibit macrophage cholesterol accumulation and prevent foam cell formation, via reduction of oxidized lipids influx, inhibition of macrophage cholesterol synthesis, and stimulation of macrophage cholesterol efflux [160].

PON2 and PON3 modes of action, despite similarities to that of PON1, have not been completely clarified. The antiatherogenic effect of PON2 could be attributed to the protection of mitochondria against oxidative stress, while PON3 has been shown to be a protective factor against obesity [15].

The PON family appears to play an important protective role in the process of the atheroma plaque formation, but pathogenic mechanisms are hard to establish [162, 163]. Genetic deletion of PON1 has been associated with increased susceptibility of LDL to oxidation ex vivo, increased measures of macrophage oxidative stress, and increased lesion size in animal models of atherosclerosis [164]. Decreased PON1 activity leads to malfunction of the HDL molecule with subsequent activation of the LOX-1 receptor (an endothelial lectin-like oxidized LDL-receptor) and endothelial PKC*β*II, followed by inhibition of eNOS-activating pathways and decreased NO production with ensuing impaired endothelial repair [165].

There is general agreement in the literature that serum PON1 paraoxonase activity is decreased in patients with coronary artery disease or acute coronary syndromes, as compared to healthy individuals [166, 167]. Lower serum PON1 activity correlates with increased plaque formation and a larger atherosclerotic burden thus probably suggesting a higher cardiovascular risk as well. Accordingly, in terms of prevention and prediction of CAD and mortality, a significant correlation was found between lower PON and arylesterase activity and an increased risk of major adverse cardiac events including death, myocardial infarction, and stroke [168, 169]. On the other hand, the association of the various PON genetic polymorphisms with coronary heart disease remains controversial due to the conflicting results which have been reported by different clinical studies [170, 171].

It is well established that reduced serum PON1 activity in CKD contributes to the increased burden of cardiovascular disease in this patient group, being more prominent in end-stage CKD. PON1 concentration and activity are lower in patients with chronic renal failure and in patients under renal replacement therapy as compared to healthy subjects whereas the effect of renal transplantation on PON1 activity remains unclear [172–176]. However, the prevalence PON1 genetic polymorphisms are not significantly different among patients with CKD and healthy individuals [175]. The accumulation of uremic toxins and AGE-s as well as alterations of HDL subclass distribution and function as it occurs in CKD has been proposed as possible culprits of the observed decreased concentration and activity of PON in renal failure [177]. In patients with CKD, decreased antioxidant activity of PON1 predicts an increased risk of adverse cardiovascular events and PON1 concentration has been prospectively associated with cardiovascular mortality and all-cause mortality in patients under renal replacement therapy [158, 178].

There are only few recent publications both experimental and clinical, regarding PON and its association with LVH and HF of either ischemic or nonischemic etiology. A novel antihypertrophic role for the PON gene cluster has been suggested by experimental data [178]. Additionally, reduced PON1 activity has been associated with increased arterial stiffness as well as increased insulin resistance with both states promoting myocardial remodeling [179]. Recent data suggest an association between PON1 and arterial hypertension with PON1 concentration being significantly lower in hypertensive patients [180, 181]. In patients with systolic HF, decreased PON activity is predictive of increased risk of long-term adverse cardiac events. HDL function is significantly impaired, and oxidation products of arachidonic and linoleic acids are markedly elevated in patients with HF compared with non-HF controls whereas the antioxidative and cholesterol efflux capacities of HDL are reduced in ischemic cardiomyopathy [182–184]. On the other hand, there is a paucity of data regarding the role of PON1 in alterations of cardiac structure induced by CKD. Accordingly there is only one study demonstrating an association between a genetic polymorphism of the PON1 gene to the severity of LVH and LV dysfunction in patients with CKD [185]. Finally, reduced PON1 activity has been significantly associated with increased arterial stiffness in renal transplant recipient [186].

## 7. Future Directions

In the setting of the hostile uremic milieu, there exists only fragile steady-state equilibrium, with only minor precipitating factors tipping the balance towards decompensation and major cardiovascular events. Thus, it cannot be overemphasized that further elucidation of the oxidative pathways involved in cardiorenal damage will provide substantial aid so as to improve prevention and treatment strategies in patients with CKD who are at highest risk for cardiovascular complications. The continuously increasing information regarding oxidative stress and its mechanisms of disease, the oxidative pathways activated in CKD, and consequent cardiovascular damage requires translation into clinical evidence-based medicine. Further, appropriately powered and meticulously designed clinical studies with long follow-up are needed to reliably assess the effects of antioxidant therapy in patients with CKD.
